# Neuroglia in the autistic brain: evidence from a preclinical model

**DOI:** 10.1186/s13229-018-0254-0

**Published:** 2018-12-27

**Authors:** Maria Rosanna Bronzuoli, Roberta Facchinetti, Davide Ingrassia, Michela Sarvadio, Sara Schiavi, Luca Steardo, Alexei Verkhratsky, Viviana Trezza, Caterina Scuderi

**Affiliations:** 1 Department of Physiology and Pharmacology, “Vittorio Erspamer” SAPIENZA University of Rome, 00185 Rome, Italy; 2 Department of Science, Section of Biomedical Sciences and Technologies, University “Roma Tre”, 00154 Rome, Italy; 3 Faculty of Biology, Medicine and Health, The University of Manchester, Manchester, M13 9PT UK; 4 Center for Basic and Translational Neuroscience, Faculty of Health and Medical Sciences, University of Copenhagen, 2200 Copenhagen, Denmark; 5 Achucarro Center for Neuroscience, IKERBASQUE, Basque Foundation for Science, 48011 Bilbao, Spain

**Keywords:** Autism spectrum disorder, Astrocyte, Microglia, Oligodendrocyte, Valproic acid

## Abstract

**Background:**

Neuroglial cells that provide homeostatic support and form defence of the nervous system contribute to all neurological disorders. We analyzed three major types of neuroglia, astrocytes, oligodendrocytes, and microglia in the brains of an animal model of autism spectrum disorder, in which rats were exposed prenatally to antiepileptic and mood stabilizer drug valproic acid; this model being of acknowledged clinical relevance.

**Methods:**

We tested the autistic-like behaviors of valproic acid-prenatally exposed male rats by performing isolation-induced ultrasonic vocalizations, the three-chamber test, and the hole board test. To account for human infancy, adolescence, and adulthood, such tasks were performed at postnatal day 13, postnatal day 35, and postnatal day 90, respectively. After sacrifice, we examined gene and protein expression of specific markers of neuroglia in hippocampus, prefrontal cortex, and cerebellum, these brain regions being associated with autism spectrum disorder pathogenesis.

**Results:**

Infant offspring of VPA-exposed dams emitted less ultrasonic vocalizations when isolated from their mothers and siblings and, in adolescence and adulthood, they showed altered sociability in the three chamber test and increased stereotypic behavior in the hole board test. Molecular analyses indicate that prenatal valproic acid exposure affects all types of neuroglia, mainly causing transcriptional modifications. The most prominent changes occur in prefrontal cortex and in the hippocampus of autistic-like animals; these changes are particularly evident during infancy and adolescence, while they appear to be mitigated in adulthood.

**Conclusions:**

Neuroglial pathological phenotype in autism spectrum disorder rat model appears to be rather mild with little signs of widespread and chronic neuroinflammation.

## Background

Autism spectrum disorder (ASD) is a heterogeneous set of neurodevelopmental disorders characterized by deficits in social communication and social interaction, stereotypies, and reduced patterns of behaviors [1, 2]. Even though ASD can be diagnosed at any age, symptoms generally appear in the childhood and last throughout a person’s life. Although about 1% of the world population suffers from ASD [3], little is known on ASD etiology and pathogenesis. Genetic predispositions, maternal stressors, environmental factors, infectious agents, and the intake of specific drugs during pregnancy all have some degree of association with ASD [4]. One of the common environmental factors involved in the pathogenesis of ASD is maternal exposure to the antiepileptic and mood stabilizer drug valproic acid (VPA). When given during pregnancy, VPA was reported to induce various congenital malformations [5, 6] including autistic-like features in the exposed children, such as impaired communication, reduced sociability and stereotyped behaviors [7, 8]. Based on these clinical observations, prenatal VPA exposure in rodents has been developed and became a widely used environmental preclinical model of ASD with face and construct validity [9–11].

Recent findings highlight contribution of neuroglia to the ASD pathophysiology. Glial cells are non-excitable homeostatic cells of the central nervous system (CNS), sub-classified into astrocytes, oligodendrocytes and their precursors (also known as NG-2 glia) and microglia; all types of glia sustain vital brain functions [12]*.* Specifically, astroglial cells are key cellular contributors to the homeostasis of the nervous tissue and the brain as an organ [13–16]. Astrocytes regulate pH and ion homeostasis, regulate functional hyperaemia and provide trophic and metabolic support to neurones. Astrocytes are important elements of the cytoarchitecture of the brain. These cells are essential for synaptogenesis [17, 18] as well as synaptic remodeling and are likely to contribute to various aspects of memory formation, storage, and retention [19]. Oligodendrocytes form the myelin sheath, thus maintaining the functional connectome of the brain and contributing to the optimal information processing in complex neural networks [20]. Microglia provide the immune and cellular defence in the brain. Through several surveillance mechanisms, microglia detect diverse pathological extracellular signals, and respond to them to protect the brain. These cells also contribute to the development of the nervous tissue, shaping neuronal ensembles and synaptic plasticity [21–24]*.*

Increasing appreciation of the multifaceted physiological roles of glia in the developing and mature CNS suggests that abnormalities in glial functions contribute to neuropathology. Several preclinical models of diseases revealed the role of glia in neurodevelopmental diseases, from ASD to neuropsychiatric disorders. Pathological changes in neuroglia are complex and can be classified into reactive response (astrogliosis, activation of microglia, and Wallerian remodeling of oligodendrocytes), degeneration with atrophy and loss of function (characteristic for astrocytes and microglia), and pathological remodeling [25–27]. The contribution of glial cells to pathological development of cognitive and neuropsychiatric disorders, such as Alzheimer’s disease, Parkinson’s disease, depression, schizophrenia, and others, has been demonstrated [28–36]. The role of glia in ASD however is not clear and often the data available are limited to their involvement in the inflammatory response.

In this study, we performed an in-depth analysis of gene and protein expression of specific markers of astrocytes, oligodendrocytes, and microglia in the rats prenatally exposed to VPA (ASD animal model). We studied brain areas critically involved in ASD, namely, hippocampus (HPC), prefrontal cortex (PfC), and cerebellum (Cb) [37–39]. To account for human infancy, adolescence, and adulthood, brain tissues were analyzed at three different ages, at postnatal day (PND) 13, PND 35, and PND 90. Our results indicate that prenatal VPA exposure affects all types of neuroglia, mainly causing transcriptional modifications. The most significant changes occur in PfC and in the HPC of autistic-like animals; these changes are particularly evident during infancy and adolescence, while they appear to be mitigated in adulthood.

## Methods

All animal procedures were performed in agreement with the guidelines of the Italian Ministry of Health (D.L. 26/2014) and with the European Parliament directive 2010/63/EU.

### Animals

**Fig. 1:**
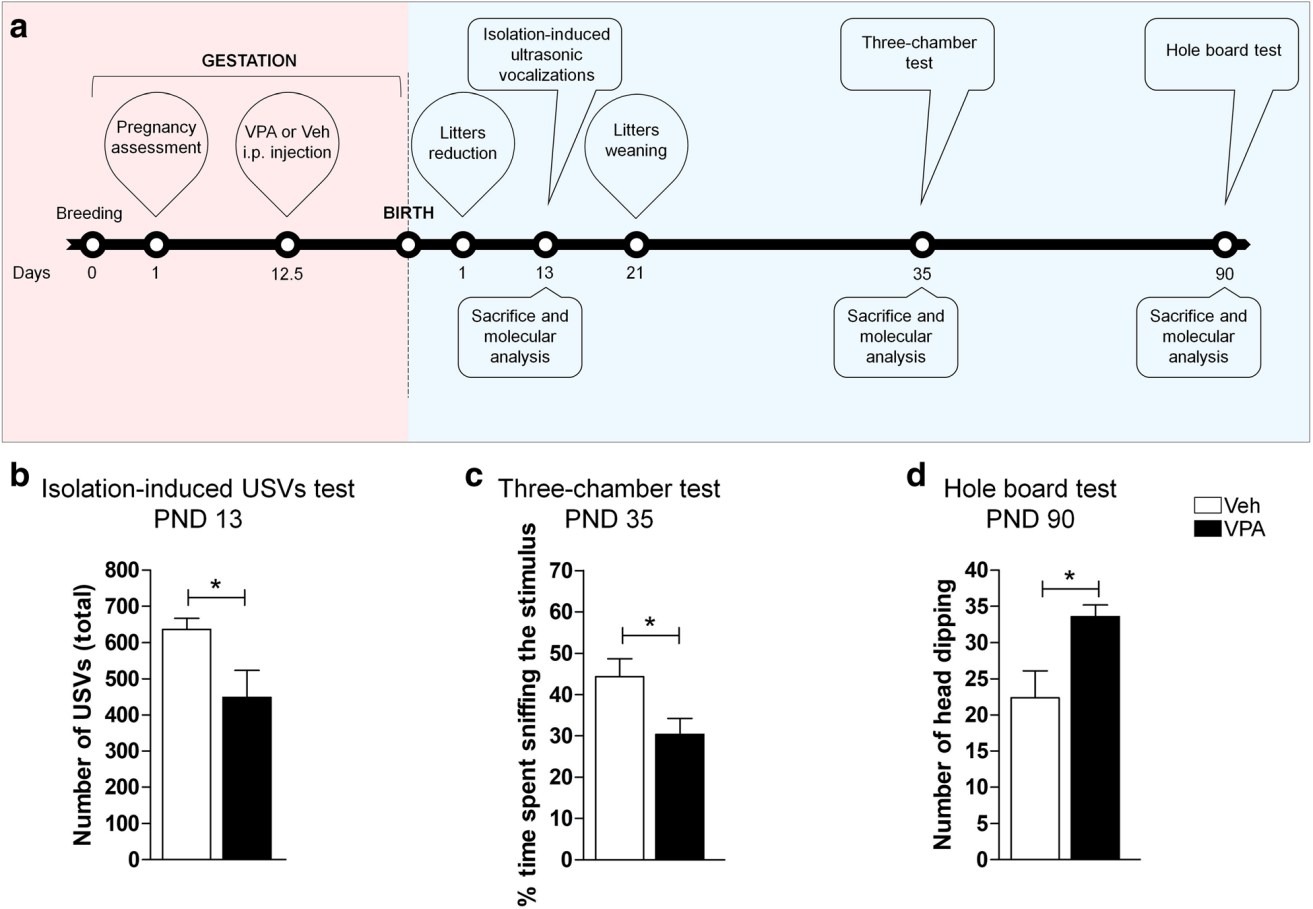
Effect of prenatal VPA exposure on animals’ behavior. Schematic representation of the experimental design (**a**). Assessment of the autistic-like phenotype in prenatally VPA-exposed rats through reduced isolation-induced USV emission at PND 13 (**b**), sociability in the three-chamber test at PND 35 (**c**), and induced stereotypic behavior in the hole-board test at PND 90 (**d**) (Veh *n* = 5, VPA *n* = 5). Data represent mean ± SEM. Statistical analysis was performed by *t* test (*p < 0.05 vs Veh group)

The offspring born from VPA-exposed dams was obtained as described previously [40]. Adult female Wistar rats (Charles River, Arbresle, France) were housed and raised under controlled conditions (22 ± 2 °C temperature, 55–65% relative humidity, 12-h light/12-h dark cycle with lights on at 07:00 h) in an enriched environment, with food and water available ad libitum. Rats weighing 250 ± 15 g were mated overnight, and the morning when spermatozoa were found was assigned as gestational day 1 (GD 1). Pregnant rats, singly placed in Macrolon cages (40 × 26 × 20 cm), on GD 12.5, received an intraperitoneal injection of either VPA (500 mg/kg in saline) or saline (Veh). This dose of VPA, administered at this developmental time point, is known to induce autistic-like traits in the exposed rat offspring at infancy, adolescence, and adulthood [41]. The day after birth (PND 1), the litters were culled to six males and two females to reduce the litter size-induced variability in the growth and development of pups during the postnatal period. However, epidemiological studies report a higher incidence of ASD in boys than in girls, and it has been shown that the autistic-like-behaviors displayed by rats prenatally exposed to VPA are more pronounced in the male than in the female offspring [42, 43]. For these reasons, only the male offspring was used in this study. After weaning on PND 21, pups were weaned and housed in groups of three. In order to perform the molecular analysis of the brains in infancy, adolescence, and adulthood, the male offspring (one rat/litter/treatment) was sacrificed on PND 13, PND 35, and PND 90, respectively. After decapitation, PfC, HPC, and Cb were rapidly isolated to perform western blot and real time-quantitative PCR (RT-qPCR); whereas, whole brains for immunofluorescence were flash-frozen in 2-methylbutane and stored at − 80 °C. The experimental design is outlined in Fig. 1a.

### Behavioral tests

#### Isolation-induced ultrasonic vocalizations (USVs)

On PND 13, the USVs emitted by each pup removed from the nest and placed into a Plexiglas arena were detected for 3 min by an ultrasound microphone (Avisoft Bioacoustics, Germany) sensitive to frequencies between 10 and 200 kHz. The USVs were analyzed quantitatively using Avisoft Recorder software (Version 5.1).

#### Three-chamber test

The test was performed as previously described [40]. The apparatus was a rectangular three-chamber box, with two lateral chambers (30 l × 35 w × 35 h cm) connected to a central chamber (15 l × 35 w × 35 h cm). Each lateral chamber contained a small Plexiglas cylindrical cage. At PND 35, each experimental rat was individually allowed to explore the three-chamber apparatus for 10 min, and then confined in the central compartment. An unfamiliar stimulus animal was confined in a cage located in one chamber of the apparatus, while the cage in the other chamber was left empty. Both doors to the side chambers were then opened, allowing the experimental animal to explore the apparatus for 10 min. The percent of time spent in social approach (sniffing the stimulus animal) were scored using the Observer 3.0 software (Noldus, The Netherlands).

#### Hole board test

The apparatus was a gray square metal table (40 l × 40 w × 10 h cm) with 16 evenly spaced holes (4 cm in diameter), inserted in a Plexiglas arena (40 l × 40 w × 60 h cm). At PND 90, rats were individually placed in the apparatus and their behavior was observed for 5 min. Dipping behavior was scored by the number of times an animal inserted its head into a hole at least up to the eye level. Each session was recorded with a camera positioned above the apparatus for subsequent behavioral analysis performed using the Observer 3.0 software (Noldus Information Technology).

### Real-time quantitative PCR (RT-qPCR)

Total mRNA of PfC, HPC, and Cb was isolated by TRI-Reagent (Sigma-Aldrich, Saint Louis, MO, USA) following the manufacturer’s instructions. For each brain tissue, the total amount of the mRNA was quantified by D30 BioPhotometer spectrophotometer (Eppendorf AG, Hamburg, Germany). The first-strand cDNA synthesis kit, adding oligo (dT) 0.2 μM and random primers 0.05 μg/μl was used to perform revers transcription of 1 μg mRNA to obtain cDNA (Promega, Promega Corporation, WI, USA). Reverse transcription was carried out with the following thermal protocol: + 25 °C for 10 min and + 72 °C for 65 min. Samples were stored at + 4 °C and then processed for mRNA encoding for S100B, glial fibrillary acidic protein (GFAP), Olig2, Iba1 (Bio-Fab laboratories, Rome, Italy), and the cluster of differentiation 11b (CD11b) (Bio-Rad, Hercules, CA, USA).

To confirm pair’s primers efficiency, the amplification products from each primer pair were tested with the melting curve analyses. The amounts of the amplicons were normalized against TATA-box binding protein (TBP) and hypoxanthine guanine phosphoribosyl transferase (HPRT) used as reference genes (all primers sequences are listed in Table 1). All amplifications were performed dissolving 500–800 nM primers and 75 ng cDNA in the iTaq Universal SYBR Green Supermix (Bio-Rad) using a CFX96 Touch thermocycler (Bio-Rad) according to the manufacturer’s instructions. The detection of the fluorescent signals was assessed at the end of the + 60 °C extension period. For each sequence of interest, three independent experiments were performed in triplicate. Data are expressed as the fold difference in mRNA expression (ΔΔCq) calculated according to the Pflaffl method.

**Table 1:** Primer sequences and general conditions used to perform real-time qPCR

GENE	Primer (5′ → 3′)		Annealing (°C)	Efficiency (%)	*R* ^2^
S100B	Forward	TCAGGGAGAGAGGGTGACAA	60	94.6	.998
	Reverse	ACACTCCCCATCCCCATCTT			
GFAP	Forward	CGGCTCTGAGAGAGATTCGC	60	105.0	.989
	Reverse	GCAAACTTGGACCGATACCA			
Olig2	Forward	CCCGATGATCTTTTTCTGCC	60	98.8	.990
	Reverse	GCTTCTTATCTTTCTTGGTG			
CD11b	Forward	N/A (Cod. qRnoCID0002800, Bio-Rad)	60	94.0	.990
	Reverse				
Iba1	Forward	GTCCTTGAAGCGAATGCTGG	60	95.6	.994
	Reverse	CATTCTCAAGATGGCAGATC			
HPRT	Forward	TCCCAGCGTCGTGATTAGTGA	60	98.3	.992
	Reverse	CCTTCATGACATCTCGAGCAAG			
TBP	Forward	TGGGATTGTACCACAGCTCCA	60	99.7	.995
	Reverse	CTCATGATGACTGCAGCAAACC			

*GFAP* glial fibrillary acidic protein; *CD11b* cluster of differentiation 11b; *HPRT* hypoxanthine guanine phosphoribosyl transferase; *TBP* TATA-box binding protein

### Western blot

Total protein amount of PfC, HPC, and Cb was isolated and processed as previously described [29, 30, 34]. Brain tissues were mechanically lysed in ice-cold hypotonic lysis buffer containing 50 mM Tris/HCl pH 7.5, 150 mM NaCl, 1 mM ethylenediamenetetraacetic acid (EDTA), 1% triton X-100, 1 mM phenylmethylsulfonyl fluoride (PMSF), 10 μg/ml aprotinin, and 0.1 mM leupeptin (all from Sigma-Aldrich), and then incubated for 40 min at + 4 °C. After centrifugation at 14000 rpm for 30 min, supernatants were collected and stored at − 80 °C. Protein concentration was calculated by Bradford assay to resolve an equal amount of proteins for each sample. Thirty micrograms were resolved through 12% acrylamide SDS-PAGE gel and then transferred onto nitrocellulose membranes with a trans-blot semi-dry transfer cell (Bio-Rad). From this step on, membranes were treated on an orbital shaker. Unspecific bound of the antibodies was avoided by incubating membranes for 1 h at room temperature in a blocking solution containing either 5% non-fat dry milk (Bio-Rad) or 5% bovine serum albumin (BSA, Sigma-Aldrich) in tris-buffered saline (TBS) (Corning, NY, USA) 0.1% tween 20 (TBS-T). Then, an overnight incubation with the proper primary antibodies against S100B, GFAP, Olig2, CD11b, or Iba1 was performed at + 4 °C (experimental conditions are reported in Table 2).

**Table 2:** Experimental conditions used to perform western blot experiments

Primary antibody	Brand/cat #	Dilution	Secondary antibody	Brand/cat #
Rabbit α-S100B	Genetex	1:1000	HRP conjugated goat anti-rabbit IgG 1:10000	Jackson ImmunoResearch
	GTX129573	5% BSA in TBS-T 0.1%	5% BSA in TBS-T 0.1%	111-035-045
Rabbit α-GFAP	Abcam	1:25000	HRP conjugated goat anti-rabbit IgG 1:10000	Jackson ImmunoResearch
	ab7260	5% milk in TBS-T 0.1%	5% milk in TBS-T 0.1%	111-035-045
Rabbit α-Olig2	Santa Cruz	1:500	HRP conjugated goat anti-rabbit IgG 1: 10000	Jackson ImmunoResearch
	sc-48817	5% milk in TBS-T 0.1%	5% milk in TBS-T 0.1%	111-035-045
Rabbit α-CD11b	Bioss	1:1000	HRP conjugated goat anti-rabbit IgG 1:10000	Jackson ImmunoResearch
	bs-1014R	5% BSA in TBS-T 0.1%	5% BSA in TBS-T 0.1%	111-035-045
Rabbit α-Iba1	Abcam	1:1000	HRP conjugated goat anti-rabbit IgG 1:10000	Jackson ImmunoResearch
	ab178846	5% milk in TBS-T 0.1%	5% milk in TBS-T 0.1%	111-035-045
Rabbit α-β-actin	Santa Cruz	1:1000	HRP conjugated goat anti-rabbit IgG 1:20000	Jackson ImmunoResearch
	sc-1616R	5% milk in TBS-T 0.1%	5% milk in TBS-T 0.1%	111-035-045

*GFAP* glial fibrillary acidic protein; *CD11b* cluster of differentiation 11b; *MAP2* microtubule associated protein; *BSA* bovine serum albumin; *TBS-T* tris buffered saline tween 20; *HRP* horseradish peroxidase

After removing the excess of antibody solution, the membranes were rinsed in TBS-T 0.05% and incubated for 1 h at room temperature with a specific secondary horseradish peroxidase (HRP)-conjugated antibody (Table 2) to detect immunocomplexes by an enhanced chemiluminescence (ECL) kit (GE Healthcare Life Sciences, Milan, Italy). Immunocomplexes were visualized using a Chemidoc XRS+ and Image Lab software (Bio-Rad), and then quantified by ImageJ software. Values were normalized to those of β-actin.

For each protein of interest, three independent experiments were performed in triplicate. Data are expressed as percentage of control.

### Immunofluorescence

Immunofluorescence was performed as previously described [30, 34, 44]. The assay was performed on 12-μm-thick coronal slices of PfC, HPC, and Cb. Tissues were rinsed in phosphate-buffered saline (PBS) and post-fixed with 4% paraformaldehyde (PFA). After the blocking step lasting 90 min at room temperature in 1% BSA dissolved in PBS/0.25% triton X-100, sections were incubated overnight with the primary antibody recognizing GFAP, Olig2, or Iba1 at + 4 °C. Primary antibodies were diluted in 0.5% BSA in PBS/0.25% triton X-100. Tissues were rinsed in PBS and incubated for 2 h at room temperature with the proper secondary antibody. The staining of nuclei was performed with Hoechst (1:5000, Thermo Fisher Scientific, MA, USA). After rinses in PBS slices were mounted with Fluoromount aqueous mounting medium (Sigma-Aldrich). The experimental conditions are summarized in Table 3.

**Table 3:** Experimental conditions used to perform immunofluorescence

Primary antibody	Brand/cat #	Dilution	Secondary antibody	Brand/cat #
Rabbit α-GFAP	Abcam	1:200	FITC conjugated goat anti-rabbit IgG (H + L) 1:200, 5% BSA in PBS/0.25% triton X-100	Jackson ImmunoResearch
	ab7260	5% BSA in PBS/0.25% triton X-100		111-095-003
Rabbit α-Olig2	Santa Cruz	1:250	FITC conjugated goat anti-rabbit IgG (H + L) 1:200, 0.5% BSA in PBS/0.25% triton X-100	Jackson ImmunoResearch
	sc-48817	0.5% BSA in PBS/0.25% triton X-100		111-095-003
Rabbit α-Iba1	Wako	1:1000	FITC conjugated goat anti-rabbit IgG (H + L) 1:200, 0.5% BSA in PBS/0.25% triton X-100	Jackson ImmunoResearch
	019-19741	0.5% BSA in PBS/0.25% triton X-100		111-095-003

*GFAP* glial fibrillary acidic protein; *MAP2* microtubule associated protein; *BSA* bovine serum albumin; *FITC* fluorescein isothiocyanate; *PBS* phosphate buffered saline

### Cell count analysis

Cells labeled with the different markers were quantified in 4 serial coronal 12 μm sections, spaced 48 μm apart, in each brain region for each animal. We used three rats per experimental group (*N* = 3 vehicle and *N* = 3 VPA) for each age, for a total of 18 rats. The brain regions analyzed were the PfC, the molecular layer (ML) and the granular cell layer (GL) of the Cb, the stratum radiatum of the Ammon’s horn 1 (CA1), CA2, CA3, and hilus of the dentate gyrus (DG) of the HPC. Nuclei were stained with Hoechst dye. Cells were identified as positive for a marker if they expressed immunoreactivity visually deemed to be above background. Images were captured using a × 20/0.50 magnification objective, and digitization was executed with a wide-field microscope (Eclipse E600; Nikon Instruments, Rome, Italy) connected to a QImaging camera with NIS-Elements BR 3.2 64-bit software. We used a 200 × 100 × 12 μm capture field of view to analyze the number of immunopositive cells within each field using the multi-point button of the Fiji Is Just ImageJ (FIJI) software. Cell count analyses, expressed as number of antibody positive cells in 2.4 × 10^5^ μm^3^ of tissue, were carried out by a blind observer.

### Statistical analysis

GraphPad Prism 6 software (GraphPad Software, San Diego, CA, USA) was used for the statistical analyses. Student’s *t* test was used to compare Veh and VPA groups. Data are presented as mean ± SEM. Differences between means were considered as significant at *p* < 0.05.

## Results

### Behavioral tests

Animals prenatally exposed to VPA showed enduring impairments in the three core symptoms of autism. At infancy, VPA-exposed pups separated from the dam and siblings vocalized significantly less compared to Veh-exposed pups (*t* = 2.334; *p* < 0.05; df = 8, Fig. 1b). At adolescence, VPA-exposed rats showed decreased sociability in the three-chamber test, since they spent less time sniffing the stimulus animal compared to Veh-exposed animals (*t* = − 2.436; *p* < 0.05; df = 8, Fig. 1c). At adulthood, VPA-exposed rats showed stereotypic behaviors in the hole board test, since they made more head dipping at PND 90 (*t* = − 2.781; *p* < 0.05; df = 8, Fig. 1d).

In their entirety, these results confirm that prenatal exposure to VPA causes the manifestation of autistic-like behaviors that persist from infancy to early adulthood.

### Astrocytes in ASD model rats

**Fig. 2:**
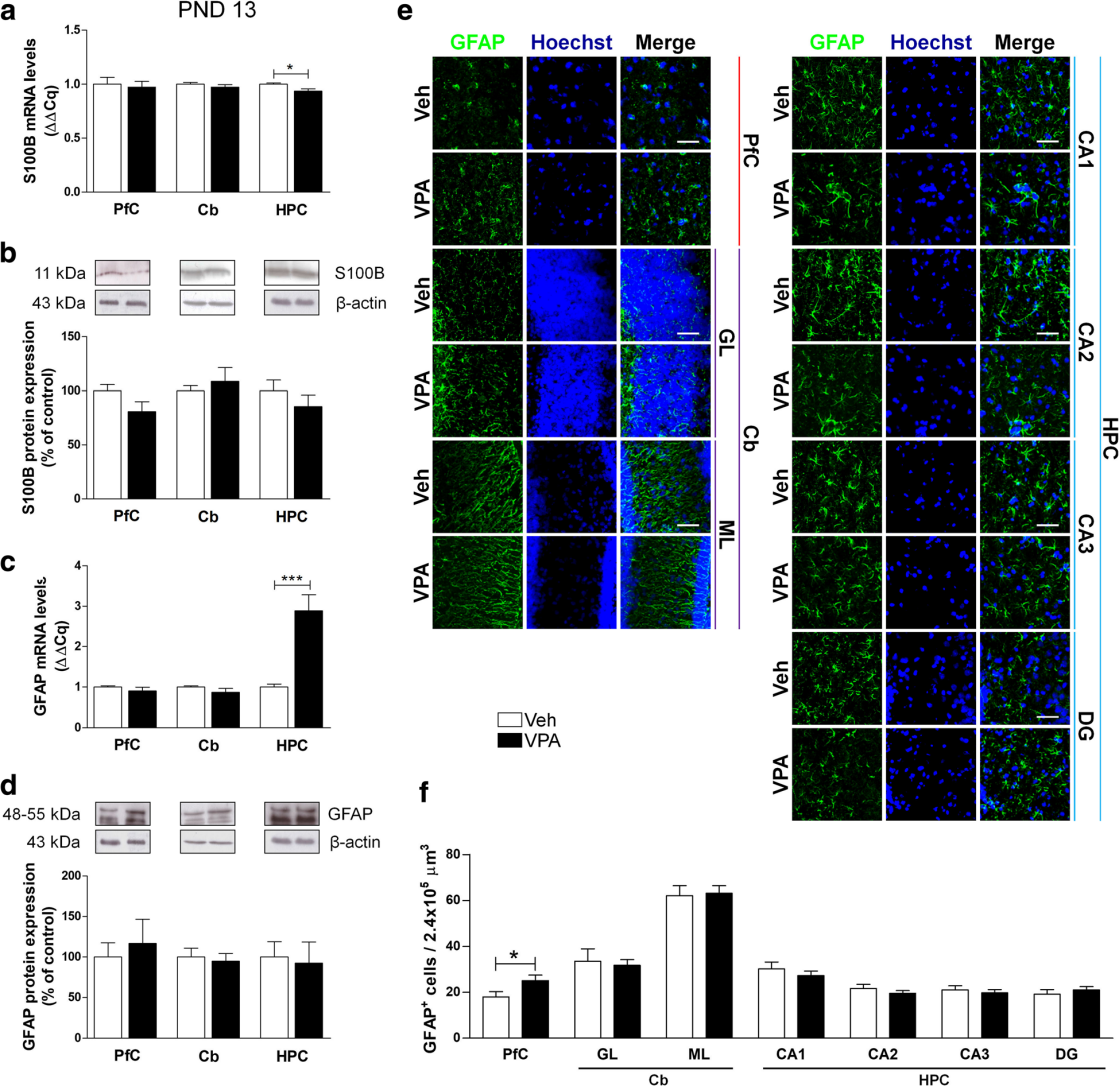
Effect of prenatal VPA exposure on astrocytes in infancy. Analysis of the neurotrophin S100B and the GFAP in the PfC, Cb, and HPC of healthy- (open bars, Veh) and autistic-like (black bars, VPA) infant rats (PND 13). Representation of the relative concentration of S100B (**a**) and GFAP (**c**) in VPA animals compared to control (Veh), normalized to both TBP and HPRT (ΔΔCq; *N* = 3, in triplicate). Representative western blots for S100B (**b**) and GFAP (**d**) proteins, and densitometric analyses are normalized to β-actin used as loading control. Results are expressed as percentage of control (Veh) (*N* = 3, in triplicate). Representative fluorescence micrographs of GFAP (green) staining in the PfCGL and MLof Cb, and CA1, CA2, CA3, and the hilus of the DG of the HPC. Nuclei were stained with Hoechst (blue) (**e**). The images have been analyzed by counting the number of GFAP-positive cells in 2.4 × 10^5^ μm^3^ (scale bar 50 μm; *N* = 3, 4 times) (**f**). All data are presented as means ± SEM. Statistical analysis was performed by *t* test (*p < 0.05; ***p < 0.001 vs Veh group)

To investigate the effect of prenatal VPA exposure on astrocyte phenotype, we analyzed transcription and expression of the archetypal astroglial markers GFAP and the neurotrophin/Ca^2+^ binding protein S100B. At PND 13, we observed a significant reduction of S100B mRNA in the HPC of VPA-exposed rats compared to control animals, with no significant modification in its protein expression (Fig. 2a, b). At the same age, we detected a significant increase of GFAP mRNA in the HPC of VPA-exposed rats (Fig. 2c). No changes in GFAP protein were observed among all groups by western blot (Fig. 2d); however, immunofluorescence experiments revealed a significant increase of GFAP-positive cells in the PfC of VPA-exposed rats (Fig. 2e, f).

**Fig. 3:**
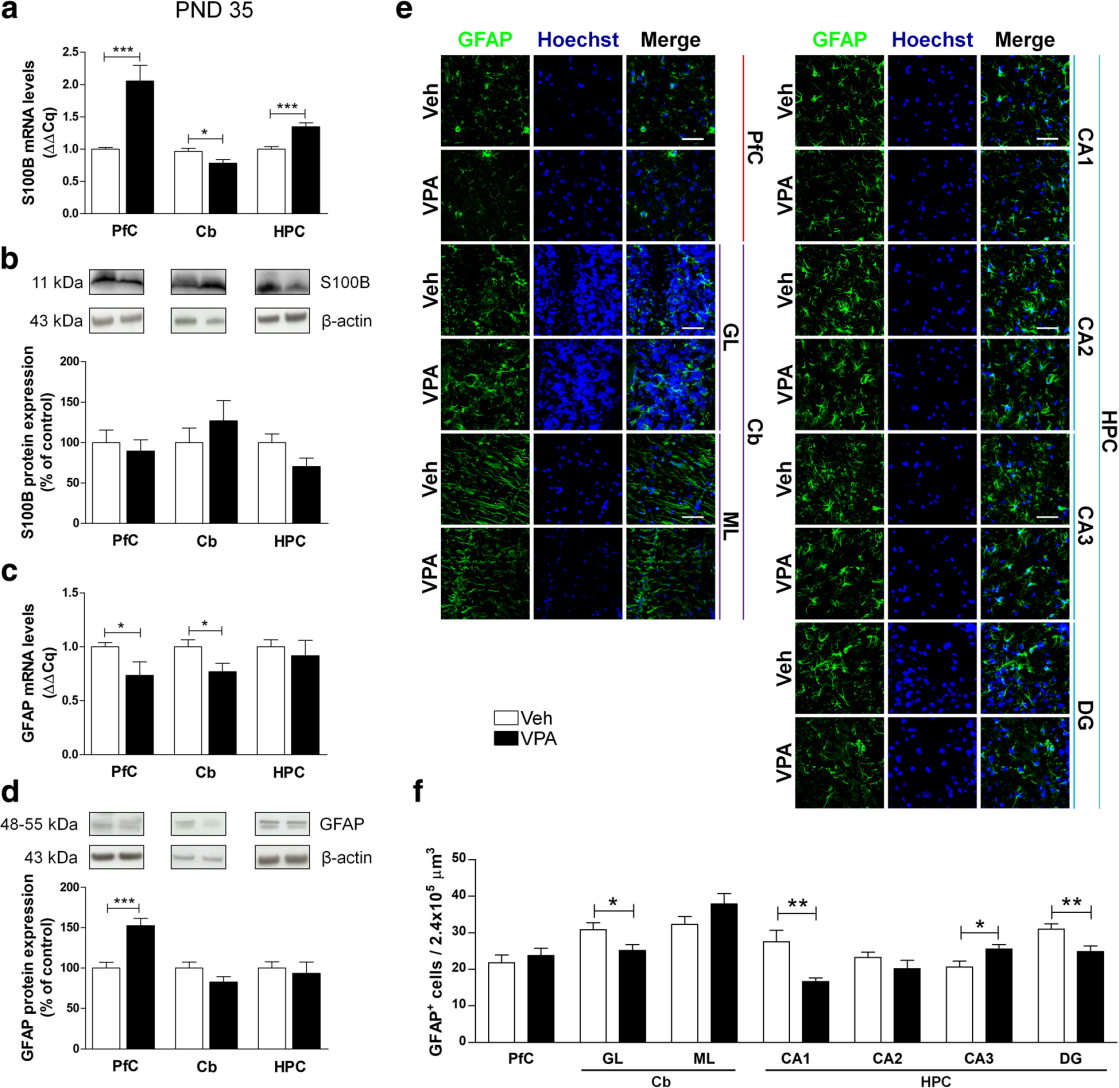
Effect of the prenatal VPA exposure on astrocytes in adolescence. Evaluation of the neurotrophin S100B and the cytoskeletal GFAP in the PfC, Cb, and HPC of healthy- (open bars, Veh) and autistic-like (black bars, VPA) adolescent rats (PND 35). Representation of the relative concentration of S100B (**a**) and GFAP (**c**) in VPA animals compared to control (Veh), normalized to both TBP and HPRT (ΔΔCq; *N* = 3, in triplicate). Representative western blots for S100B (**b**) and GFAP (**d**) proteins, and densitometric analyses normalized to β-actin used as loading control. Results are expressed as percentage of control (Veh) (*N* = 3, in triplicate). Representative fluorescence micrographs of GFAP (green) staining in the PfC, GL and ML of Cb, and stratum radiatum of CA1, CA2, CA3, and the hilus of the DG of the HPC. Nuclei were stained with Hoechst (blue) (**e**). The images have been analyzed by counting the number of GFAP-positive cells in 2.4 × 10^5^ μm^3^ (scale bar 50 μm; *N* = 3, 4 times) (**f**). All data are presented as means ± SEM. Statistical analysis was performed by *t* test (*p < 0.05; **p < 0.01; ***p < 0.001 vs Veh group)

**Fig. 4:**
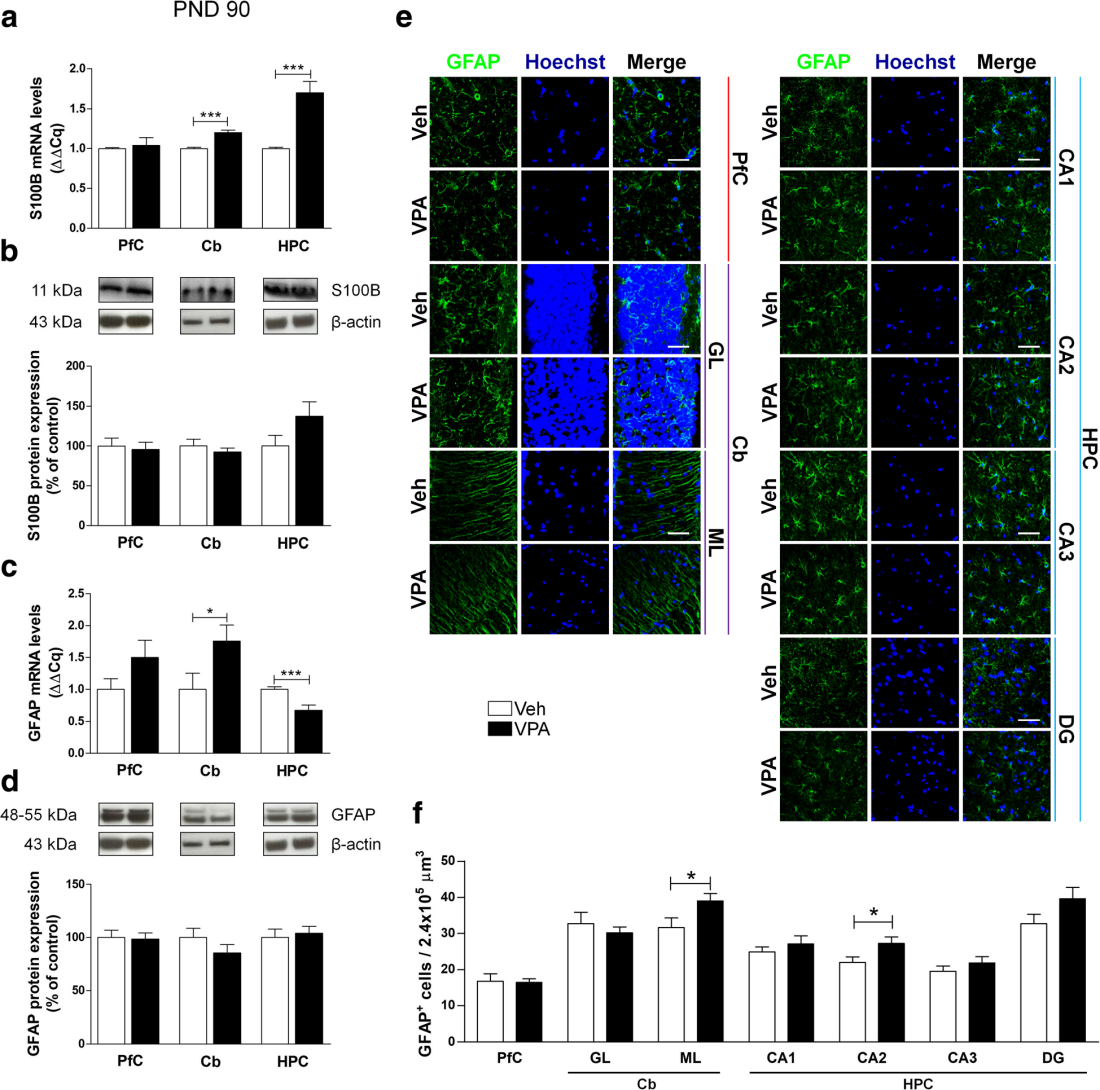
Effect of the prenatal VPA exposure on astrocytes in adulthood. Evaluation of the neurotrophin S100B and the cytoskeletal GFAP in the PfC, Cb, and HPC of healthy- (open bars, Veh) and autistic-like (black bars, VPA) adult rats (PND 90). Representation of the relative concentration of S100B (**a**) and GFAP (**c**) in VPA animals compared to control (Veh), normalized to both TBP and HPRT (ΔΔCq; *N* = 3, in triplicate). Representative western blots for S100B (**b**) and GFAP (**d**) proteins, and densitometric analyses normalized to β-actin used as loading control. Results are expressed as percentage of control (Veh) (*N* = 3, in triplicate). Representative fluorescence micrographs of GFAP (green) staining in the PfC, GL and ML of Cb, and stratum radiatum of CA1, CA2, CA3, and the hilus of the DG of the HPC. Nuclei were stained with Hoechst (blue) (**e**). The images have been analyzed by counting the number of GFAP-positive cells in 2.4 × 10^5^ μm^3^ (scale bar 50 μm; *N* = 3, 4 times) (**f**). All data are presented as means ± SEM. Statistical analysis was performed by *t* test (*p < 0.05; ***p < 0.001 vs Veh group)

At PND 35 rats showed higher levels of S100B mRNA in both PfC and HPC of VPA-exposed rats, with a significant reduction in the Cb (Fig. 3a). A decreased level of GFAP mRNA was found in the PfC and in the Cb of VPA animals compared to controls (Fig. 3c). No modifications of S100B levels were found at protein level (Fig. 3b), whereas GFAP protein expression was higher in the PfC of VPA-exposed rats (Fig. 3d).The number of GFAP-positive cells was decreased in the GL of the Cb, and in the CA1 and DG hippocampal sub-regions, while a significant increase of GFAP-positive astrocytes was observed in the CA3 of VPA-exposed rats (Fig. 3e, f). Results obtained in adult rats demonstrate transcriptional modifications and some alterations in protein content. At PND 90, VPA-exposed animals showed higher levels of S100B mRNA in Cb and HPC (Fig. 4a). Conversely, GFAP mRNA was lower in the HPC and higher in the Cb of VPA-exposed rats compared to Veh animals (Fig. 4c). No changes in the protein expression of GFAP and S100B were detected (Fig. 4b, d). Finally, significantly higher number of GFAP-positive cells in the ML of the Cb and CA2 of the HPC of VPA-exposed rats was documented (Fig. 4e, f).

In summary, prenatal exposure to VPA differentially affects astrocytes in different brain regions, and causes transcriptional modifications of S100B and GFAP, which are particularly evident in adolescent and adult rats, where modified GFAP expression is also observed.

### Oligodendrocytes in ASD model rats

**Fig. 5:**
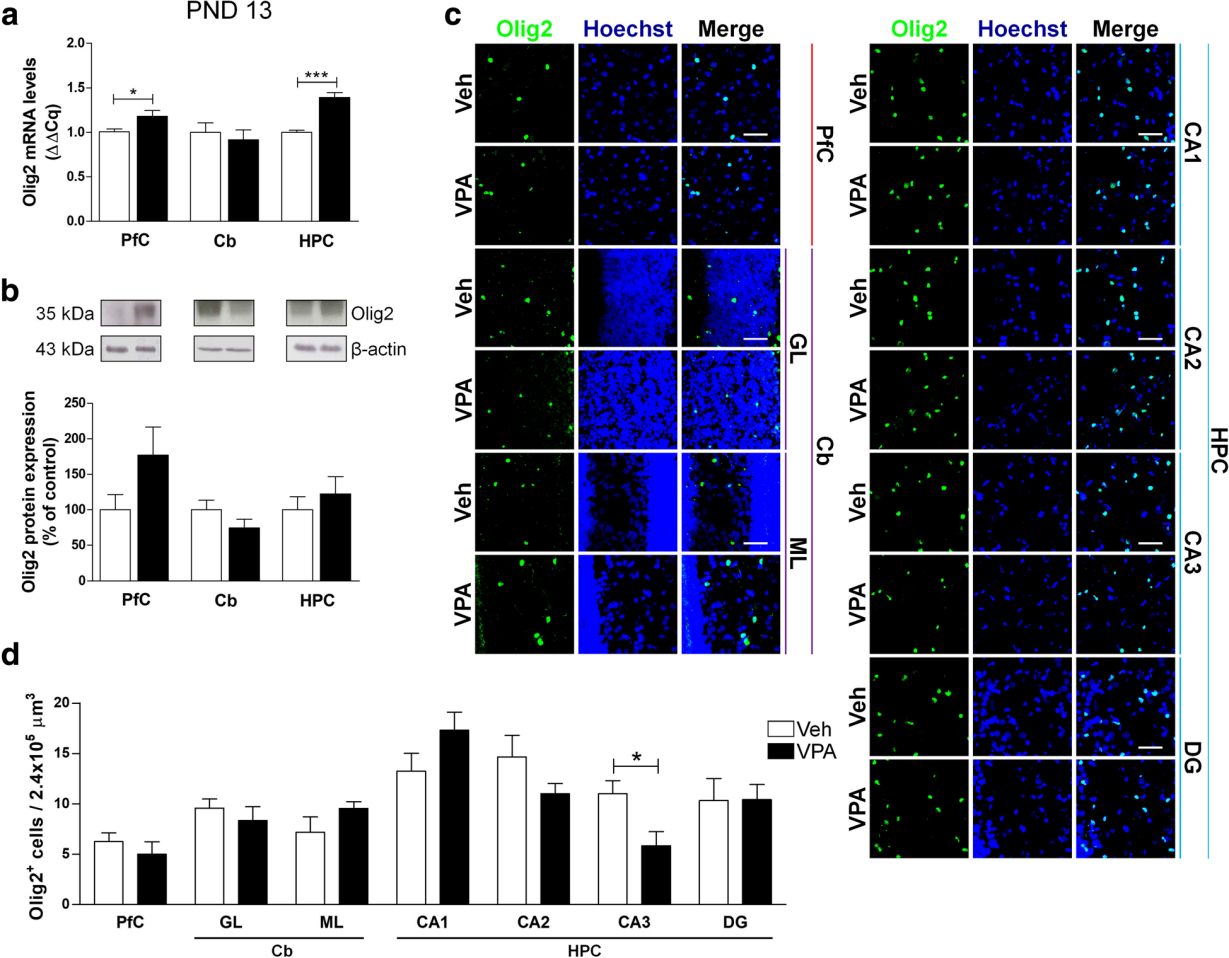
Effect of the prenatal VPA exposure on oligodendrocytes in infancy. Evaluation of the transcription factor Olig2 in the PfC, Cb, and HPC of healthy- (open bars, Veh) and autistic-like (black bars, VPA) infant rats (PND 13). Representation of the relative concentration of Olig2 (**a**) in VPA animals compared to control (Veh), normalized to both TBP and HPRT (ΔΔCq; *N* = 3, in triplicate). Representative western blots for Olig2 protein and densitometric analyses normalized to β-actin used as loading control (**b**). Results are expressed as percentage of control (Veh) (*N* = 3, in triplicate). Representative fluorescence micrographs of Olig2 (green) staining in the PfC, GL and ML of Cb, and stratum radiatum of CA1, CA2, CA3, and the hilus of the DG of the HPC. Nuclei were stained with Hoechst (blue) (**c**). The images have been analyzed by counting the number of Olig2-positive cells in 2.4 × 10^5^ μm^3^ (scale bar 50 μm; *N* = 3, 4 times) (**d**). All data are presented as means ± SEM. Statistical analysis was performed by *t* test (*p < 0.05; ***p < 0.001 vs Veh group)

We examined the effects of prenatal exposure to VPA on oligodendrocytes by testing Olig2, a transcriptional factor essential for oligodendrocyte development. Infant VPA-exposed rats showed higher levels of Olig2 mRNA in PfC and HPC, and a trend toward an increase of Olig2 protein expression (+ 76.77%) in PfC compared to age-matched control animals (Fig. 5a, b). No changes of Olig2-positive cells density were observed, except for the CA3 sub-region of the HPC where a significant decrease of their population was detected (Fig. 5c, d).

**Fig. 6:**
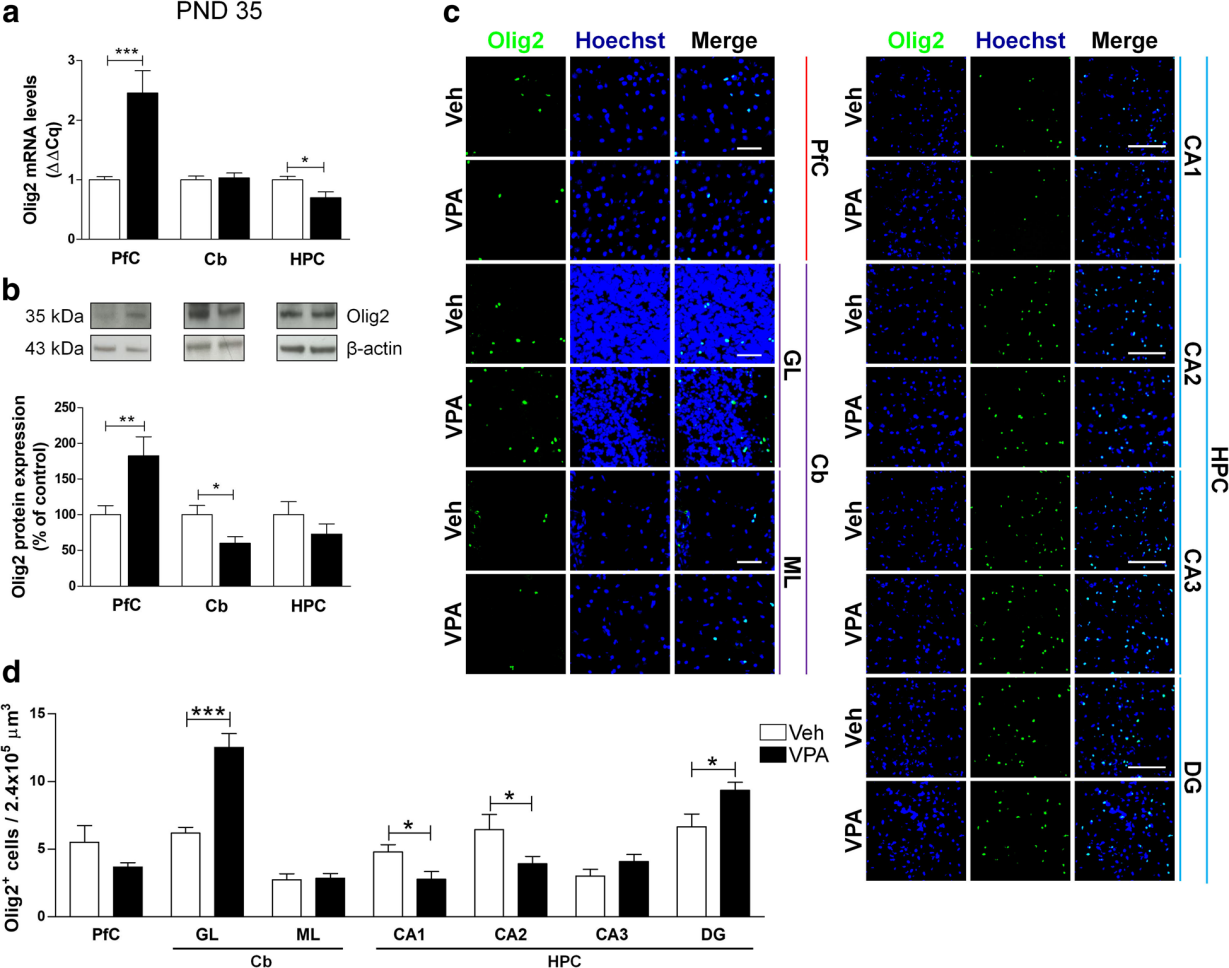
Effect of the prenatal VPA exposure on oligodendrocytes in adolescence. Evaluation of the transcription factor Olig2 in the PfC, Cb, and HPC of healthy- (open bars, Veh) and autistic-like (black bars, VPA) adolescent rats (PND 35). Representation of the relative concentration of Olig2 (**a**) in VPA animals compared to control (Veh), normalized to both TBP and HPRT (ΔΔCq; *N* = 3, in triplicate). Representative western blots for Olig2 protein and densitometric analyses normalized to β-actin used as loading control (**b**). Results are expressed as percentage of control (Veh) (*N* = 3, in triplicate). Representative fluorescence micrographs of Olig2 (green) staining in the PfC, GL and ML of Cb, and stratum radiatum of CA1, CA2, CA3, and the hilus of the DG of the HPC. Nuclei were stained with Hoechst (blue) (**c**). The images have been analyzed by counting the number of Olig2-positive cells in 2.4 × 10^5^ μm^3^ (scale bar 50 μm; *N* = 3, 4 times) (**d**). All data are presented as means ± SEM. Statistical analysis was performed by *t* test (*p < 0.05; **p < 0.01; ***p < 0.001 vs Veh group)

**Fig. 7:**
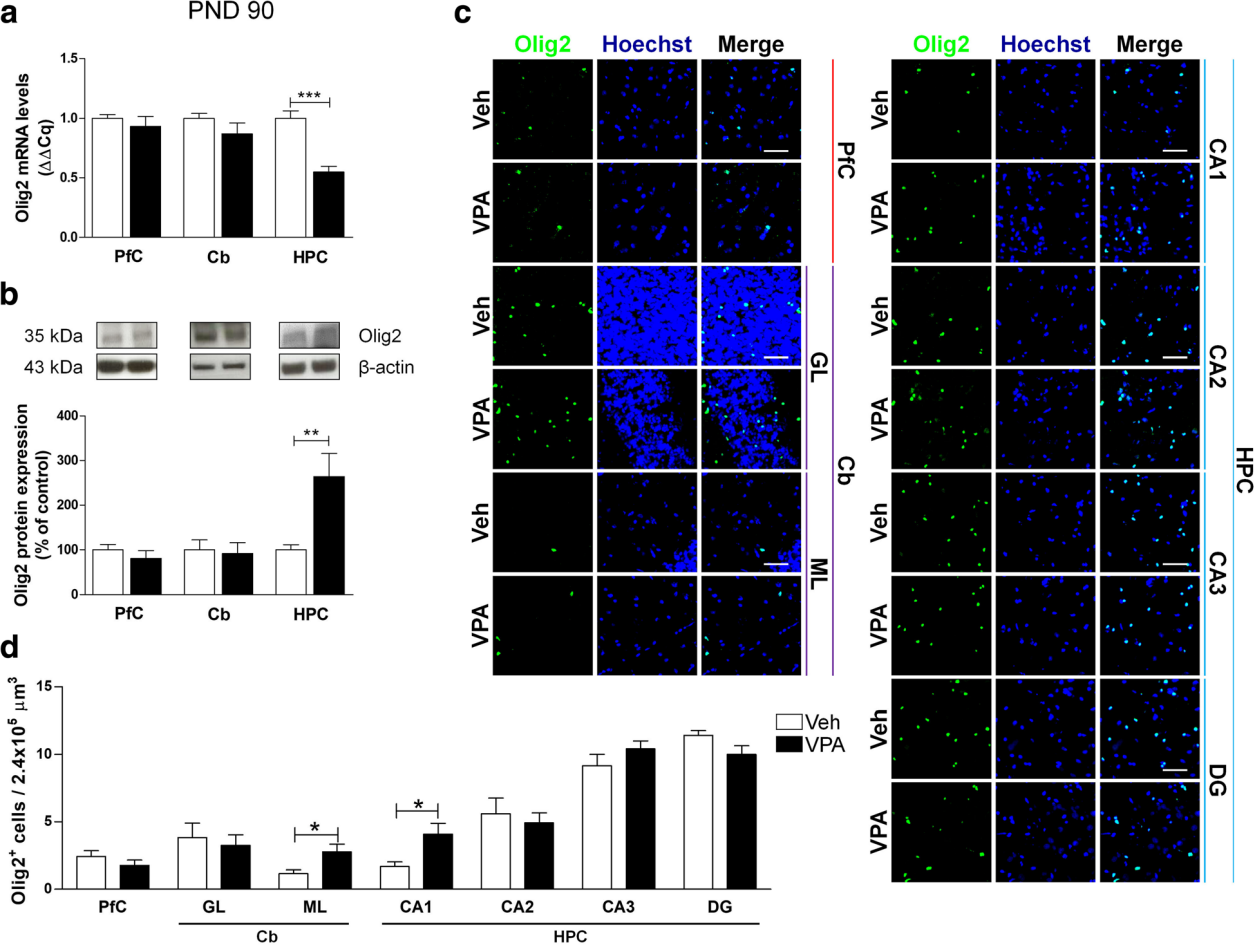
Effect of the prenatal VPA exposure on oligodendrocytes in adulthood. Evaluation of the transcription factor Olig2 in the PfC, Cb, and HPC of healthy- (open bars, Veh) and autistic-like (black bars, VPA) adult rats (PND 90). Representation of the relative concentration of Olig2 (**a**) in VPA animals compared to control (Veh), normalized to both TBP and HPRT (ΔΔCq; *N* = 3, in triplicate). Representative western blots for Olig2 protein and densitometric analyses normalized to β-actin used as loading control (**b**). Results are expressed as percentage of control (Veh) (*N* = 3, in triplicate). Representative fluorescence micrographs of Olig2 (green) staining in the PfC, GL and ML of Cb, and stratum radiatum of CA1, CA2, CA3, and the hilus of the DG of the HPC. Nuclei were stained with Hoechst (blue) (**c**). The images have been analyzed by counting the number of Olig2-positive cells in 2.4 × 10^5^ μm^3^(scale bar 50 μm; *N* = 3, 4 times) (**d**). All data are presented as means ± SEM. Statistical analysis was performed by *t* test (*p < 0.05; **p < 0.01; ***p < 0.001 vs Veh group)

The RT-qPCR analysis revealed a significant increase of Olig2 in the PfC of VPA-exposed rats at PND 35, with a decrease of this transcription factor in the HPC of the same animals (Fig. 6a). These modifications were evident also at a protein level. In adolescent VPA-exposed rats, we observed a statistically significant increase of Olig2 protein expression in the PfC, a significant decrease in the Cb, and a trend toward a decrease in the HPC (− 27.24%) (Fig. 6b). A more detailed analysis of the brain areas revealed subtler modifications in Olig2-positive cells distribution. In particular, in VPA-exposed rats we observed a significant increase in the number of Olig2-positive cells in the GL of the Cb and in the DG of the HPC, and a statistically significant reduction of Olig2-positive cells in the CA1 and CA2 of the same animals (Fig. 6c, d). In adult (PND 90) rats prenatally exposed to VPA, a significant decrease of Olig2 mRNA was observed solely in the HPC (Fig. 7a). On the contrary, the Olig2 protein was increased in the HPC of these animals (Fig. 7b). The VPA-exposed rats also showed more Olig2-positive cells in the ML of the Cb and in the CA1 (Fig. 7c, d).

Collectively, these results demonstrate that the prenatal exposure to VPA modifies oligodendrocytes at both the transcriptional and translational levels, and that these changes occur mainly in the PfC and in the HPC. Of note, these alterations are particularly evident during adolescence, but seem to be compensated in adulthood.

### Microglia in ASD model rats

**Fig. 8:**
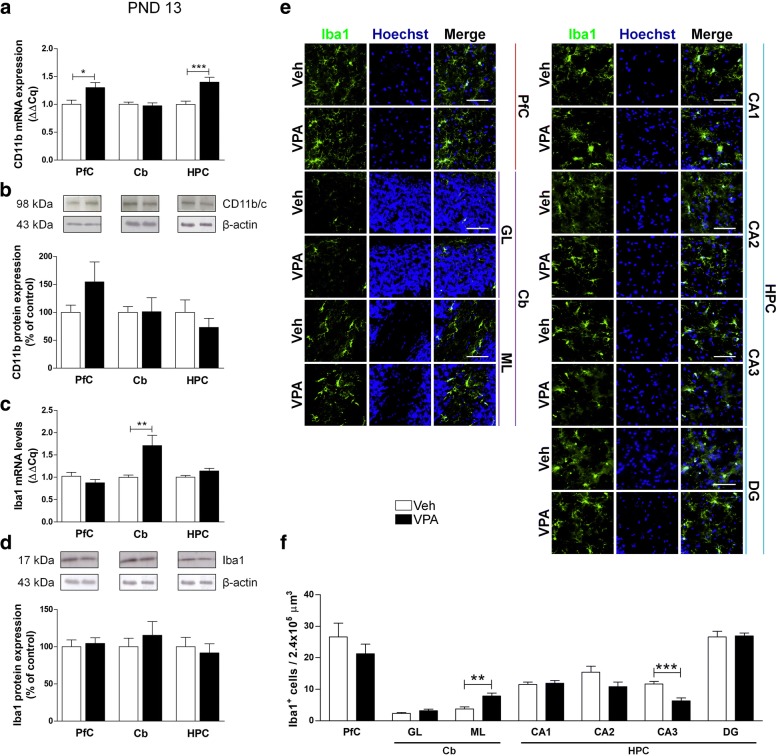
Effect of the prenatal VPA exposure on microglia in infancy. Evaluation of the cluster of differentiation 11b (CD11b) and Iba1 in the PfC, Cb, and HPC of healthy- (open bars, Veh) and autistic-like (black bars, VPA) infant rats (PND 13). Representation of the relative concentration of CD11b (**a**) and Iba1 (**c**) in VPA animals compared to control (Veh), normalized to both TBP and HPRT (ΔΔCq; *N* = 3, in triplicate). Representative western blots for CD11b (**b**) and Iba1 (**d**) proteins, and densitometric analyses normalized to β-actin used as loading control. Results are expressed as percentage of control (Veh) (*N* = 3, in triplicate). Representative fluorescence micrographs of Iba1 (green) staining in the PfC, GL and ML of Cb, and stratum radiatum of CA1, CA2, CA3, and the hilus of the DG of the HPC. Nuclei were stained with Hoechst (blue) (**e**). The images have been analyzed by counting the number of Iba1-positive cells in 2.4 × 10^5^ μm^3^ (scale bar 50 μm; *N* = 3, 4 times) (**f**). All data are presented as means ± SEM. Statistical analysis was performed by *t* test (*p < 0.05; **p < 0.01; ***p < 0.001 vs Veh group)

To characterize microglia in this rat model of ASD, we analyzed transcription and expression of CD11b, a marker of microglia activation, and Iba1, a Ca^2+^-binding protein constitutively expressed by both surveillant and activated microglia. In VPA-exposed rats at PND 13, we observed a significant increase of CD11b mRNA in PfC and HPC, and no substantial change in the protein expression except for a trend toward an increase of this marker in PfC (+ 54.36%) (Fig. 8a, b). At the same age, we detected a significant increase of Iba1 mRNA in the Cb of VPA-exposed rats (Fig. 8c). We also found a significant increase in the number of Iba-positive cells in the ML of the Cb of VPA-exposed rats with a significant decrease in the CA3 of the same animals (Fig. 8e, f).

**Fig. 9:**
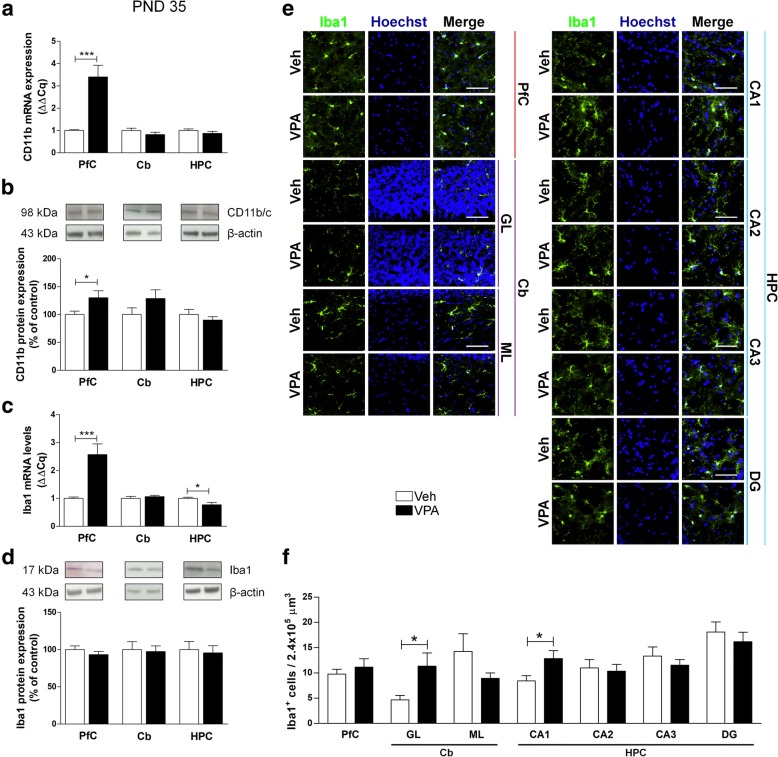
Effect of the prenatal VPA exposure on microglia in adolescence. Evaluation of the cluster of differentiation 11b (CD11b) and Iba1 in the PfC, Cb, and HPC of healthy- (open bars, Veh) and autistic-like (black bars, VPA) adolescent rats (PND 35). Representation of the relative concentration of CD11b (**a**) and Iba1 (**c**) in VPA animals compared to control (Veh), normalized to both TBP and HPRT (ΔΔCq; *N* = 3, in triplicate). Representative western blots for CD11b (**b**) and Iba1 (**d**) proteins, and densitometric analyses normalized to β-actin used as loading control. Results are expressed as percentage of control (Veh) (*N* = 3, in triplicate). Representative fluorescence micrographs of Iba1 (green) staining in the PfC, GL and ML of Cb, and stratum radiatum of CA1, CA2, CA3, and the hilus of the DG of the HPC. Nuclei were stained with Hoechst (blue) (**e**). The images have been analyzed by counting the number of Iba1-positive cells in 2.4 × 10^5^ μm^3^ (scale bar 50 μm; *N* = 3, 4 times) (**f**). All data are presented as means ± SEM. Statistical analysis was performed by *t* test (*p < 0.05; **p < 0.01; ***p < 0.001 vs Veh group)

Adolescent (PND 35) rats displayed more pronounced modifications. A significant increase of both transcription and expression of CD11b in the PfC of VPA-exposed animals compared to control rats was detected (Fig. 9a, b). Moreover, a significant increase of Iba1 mRNA was found in the PfC of VPA-exposed rats, whereas, in the same animals, we observed reduced transcription in the HPC (Fig. 9c). No changes of protein expression of Iba1 were observed (Fig. 9d). The number of Iba1-positive cells increased in the GL of the Cb and in the CA1 of adolescent (PND 35) VPA-exposed rats (Fig. 9e, f).

**Fig. 10:**
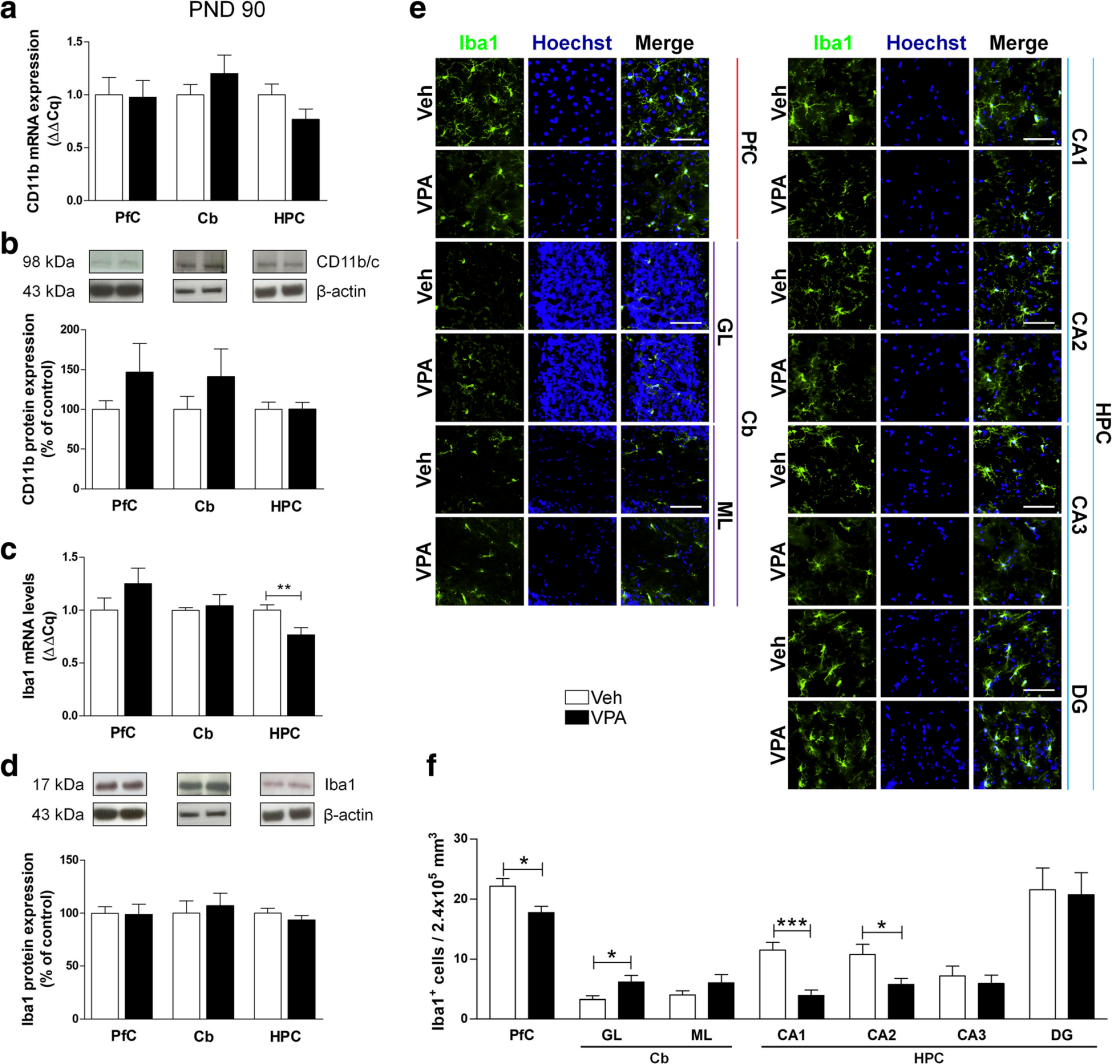
Effect of the prenatal exposure to VPA on microglia in adulthood. Evaluation of the cluster of differentiation 11b (CD11b) and Iba1 in the PfC, Cb, and HPC of healthy- (open bars, Veh) and autistic-like (black bars, VPA) adult rats (PND 90). Representation of the relative concentration of CD11b (**a**) and Iba1 (**c**) in VPA animals compared to control (Veh), normalized to both TBP and HPRT (ΔΔCq; *N* = 3, in triplicate). Representative western blots for CD11b (**b**) and Iba1 (**d**) proteins, and densitometric analyses normalized to β-actin used as loading control. Results are expressed as percentage of control (Veh) (*N* = 3, in triplicate). Representative fluorescence micrographs of Iba1 (green) staining in the PfC, GL and ML of Cb, and stratum radiatum of CA1, CA2, CA3, and the hilus of the DG of the HPC. Nuclei were stained with Hoechst (blue) (**e**). The images have been analyzed by counting the number of Iba1-positive cells in 2.4 × 10^5^ μm^3^ (scale bar 50 μm; *N* = 3, 4 times) (**f**). All data are presented as means ± SEM. Statistical analysis was performed by *t* test (*p < 0.05; **p < 0.01; ***p < 0.001 vs Veh group)

In adult rats (PND 90), no modifications of CD11b and Iba1 protein transcription and expression was observed, except for a trend toward an increase of CD11b expression in PfC (+ 47.04%) and Cb (+ 41.26%), and a significant decrease of Iba1 mRNA in the HPC of the same animals (Fig. 10a–d). At PND 90, the number of Iba1-positive cells was significantly reduced in PfC, CA1, and CA2 of rats prenatally exposed to VPA, while more Iba1-positive cells were detected in the GL of the Cb of the same animals (Fig. 10e, f).

Taken together, these results indicate that the prenatal VPA exposure modifies microglia and that these changes occur mainly in the PfC and in the HPC. Moreover, we observed that the prenatal VPA exposure switches microglial phenotypes from resting to activated in infant and adolescent rats while this phenomenon is somewhat mitigated in adulthood.

## Discussion

All three types of neuroglia are critically important for normal development of the CNS and for formation of neuronal ensembles. Astrocytes assist synaptogenesis [17, 18], while astrocytes together with microglia shape neuronal networks through synaptic pruning and removal of redundant synaptic contacts [45–47]. Functional insufficiency of neuroglia leads to neurodevelopmental pathologies [48, 49]. The role of neuroglial components in ASD has received much attention recently, when several lines of evidence have demonstrated glia-specific alterations in animal models of ASD as well as in patients suffering from this disorder (for recent reviews see [48, 49]). The transcriptome analysis of the brains of ASD patients identified significant association of the pathology with genes linked to reactive gliosis and neuroinflammation [50]. Increased expression of astroglia-specific proteins aquaporin-4 and connexin43 has been found in the autistic human tissue [51]; increase in GFAP expression and astroglial hypertrophy was also observed with cerebellum demonstrating most prominent changes [52]. Microglial activation and increase in pro-inflammatory factors were other characteristic features of ASD brain tissue [52–54].

These findings support the notion of glia-related pathological developments that may exacerbate or even drive ASD evolution. Indeed, the autistic brain is affected already at the early developmental stages, when loss of function of microglia and astrocytes can affect formation of synaptically connected neuronal networks. Equally important could be the contribution of oligodendrocytes, which shape the brain connectome. Expression of specific markers associated with cells of oligodendroglial lineage (including for example oligodendrocyte transcription factor 1/2 or myelin basic protein) is increased in the cerebella of autistic patients [55]. The single nucleotide polymorphisms of the central oligodendroglial differentiation regulator gene DUSP15 were identified in the brains of ASD patients [56], while many components of a molecular network associated with ASD are specifically enriched in oligodendroglia and white matter [57]. Changes in oligodendroglia and hence changes in white matter may be linked to a rather characteristic ASD-associated increase in the brain size (see for examples [58, 59]).

Astroglial as well as microglial abnormalities have been detected in animal models of several types of ASD associated with expression of pathologically modified genes; these include the Rett syndrome, fragile X syndrome, and tuberous sclerosis. In the Rett syndrome that arises from loss-of-function mutations in the X-linked MeCP2 encoding methyl-CpG-binding protein 2, the glial pathological phenotype has been clearly revealed. Microglial cells lacking MeCP2 triggered excitotoxicity through excessive release of glutamate [60]; whereas MeCP2-deficient astrocytes lost their ability to support neuronal growth and dendritic ramifications in vitro [61]. In the fragile X syndrome (which results from the loss of Fmr1 gene function), increased astroglial reactivity has been observed (in mice with genetic deletion of Fmr1 gene) [62].

To summarize, the gliopathology in the ASD context is mainly represented by glial reactivity, which further highlights the contribution of neuroinflammation with both processes apparently having pathological significance. In this context, we asked ourselves whether the same reactive changes are pronounced in a rodent model of ASD resulting from in utero exposure to VPA, a widely used antiepileptic drug. The use of VPA has clinical significance as indeed VPA treatment during pregnancy has been related with a higher risk of ASD in the exposed children [7, 8]. Despite this evidence, recent epidemiological studies show that the public awareness of such an association is still limited [63].

Rodents prenatally exposed to VPA are widely used as a preclinical model of ASD [9, 11, 64]. The VPA-treated animals display several ASD-like symptoms in the course of development. These animals show impairment of the communicative capabilities, alteration of the social repertoire, stereotypical behavior, and anxiety [40, 65]. In particular, in line with previous studies [66, 67], we found that the infant male offspring born from VPA-treated rats exhibit reduced ability to interact with their mothers, since they emit less ultrasonic vocalizations when isolated from their mothers and siblings. This feature is accompanied by the inability of VPA-exposed pups to recognize familiar from unfamiliar odors, this being an early sign of the impairment in social recognition [66, 68, 69]. All these aspects negatively affect the social postnatal development of the VPA-exposed offspring and persist through adolescence and adulthood [40]. Indeed, VPA-exposed rats showed altered sociability in the three chamber test and increased stereotypic behavior in the hole board test. Our results are in agreement with those obtained by other researchers showing that a single injection of VPA to pregnant rats [65, 68, 70, 71] or mice [66, 72] on gestational day 12.5 yielded offspring with a behavioral pattern strikingly similar to that observed in autism. Beside behavioral alterations, rats exposed to VPA in utero demonstrate molecular and metabolic abnormalities. Very recent experiments demonstrated that VPA exposure impairs repair of DNA damage [41], modifies cholesterol/isoprenoid metabolism, and reduces the number of oligodendrocytes leading to lower myelin and cholesterol levels in the HPC of adolescent VPA-exposed rats [44].

## Conclusions

Here, we extend this scenario by showing that prenatal VPA exposure induces autistic-like behaviors and does affect neuroglia. Modifications identified are brain region- and age-dependent. The changes in glia which we observed in VPA-exposed rats have been rather modest and occurred mostly at young ages; moreover, the changes were quite heterogeneous as they differ between brain regions, and often we have not seen obvious correlation between expression of mRNA and respective protein. In line with previous findings [24, 71], some hints for astrogliotic response were found in young animals, in which the density of GFAP-positive astrocytes has increased in the cortical regions. This increase went in parallel with elevated GFAP mRNA, without however changes in protein content. In adult rats, the number of GFAP-positive astrocytes was increased in CA3 but decreased in CA1 region and in cerebellum. Finally, in the mature rats, numbers of GFAP-positive cells were increased in Cb and CA2 hippocampal area, with no changes in expression of GFAP and S100B at a protein level. At the same time, the changes in expression of neuroglial markers seem to be rather mild, with neuroinflammatory phenotype being present mainly in young ages and being ameliorated in adulthood.

All in all, the results we obtained in VPA-exposed rats are heterogeneous and intricate and reflect the complexity of the molecular and cellular mechanisms underlying ASD. Indeed, autism is a complex disease, whose clinic features are multifaceted and intricate like equally complex and subtle should be the molecular changes causing these phenotypes.

## Abbreviations

ASD: Autism spectrum disorder
BSA: Bovine serum albumin
Cb: Cerebellum
CD11b: Cluster of differentiation 11b
CNS: Central nervous system
DG: Dentate gyrus
ECL: Enhanced chemiluminescence
EDTA: Ethylenediaminetetraacetic acid
GD: Gestational day
GFAP: Glial fibrillary acidic protein
GL: Granular layer
HPC: Hippocampus
HPRT: Hypoxanthine guanine phosphoribosyl transferase
HRP: Secondary horseradish peroxidase
ML: Molecular layer
PBS: Phosphate-buffered saline
PFA: Paraformaldehyde
PfC: Prefrontal cortex
PMSF: Phenylmethylsulfonyl fluoride
PND: Postnatal day
RT-qPCR: Real-time quantitative PCR
TBP: TATA-box binding protein
TBS-T: Tris-buffered saline 0.1% tween 20
USVs: Isolation-induced ultrasonic vocalizations
Veh: Saline
VPA: Valproic acid
